# Contribution to HIV Prevention and Treatment by Antibody-Mediated Effector Function and Advances in Broadly Neutralizing Antibody Delivery by Vectored Immunoprophylaxis

**DOI:** 10.3389/fimmu.2021.734304

**Published:** 2021-09-15

**Authors:** Meredith Phelps, Alejandro Benjamin Balazs

**Affiliations:** Ragon Institute of MGH, MIT and Harvard, Cambridge, MA, United States

**Keywords:** vectored immunoprophylaxis, HIV, broadly neutralizing antibody, VRC07, humanized mice, AAV, Fc receptor, innate immunity

## Abstract

HIV-1 broadly neutralizing antibodies (bNAbs) targeting the viral envelope have shown significant promise in both HIV prevention and viral clearance, including pivotal results against sensitive strains in the recent Antibody Mediated Prevention (AMP) trial. Studies of bNAb passive transfer in infected patients have demonstrated transient reduction of viral load at high concentrations that rebounds as bNAb is cleared from circulation. While neutralization is a crucial component of therapeutic efficacy, numerous studies have demonstrated that bNAbs can also mediate effector functions, such as antibody-dependent cellular cytotoxicity (ADCC), antibody-dependent cellular phagocytosis (ADCP), and antibody-dependent complement deposition (ADCD). These functions have been shown to contribute towards protection in several models of HIV acquisition and in viral clearance during chronic infection, however the role of target epitope in facilitating these functions, as well as the contribution of individual innate functions in protection and viral clearance remain areas of active investigation. Despite their potential, the transient nature of antibody passive transfer limits the widespread use of bNAbs. To overcome this, we and others have demonstrated vectored antibody delivery capable of yielding long-lasting expression of bNAbs *in vivo*. Two clinical trials have shown that adeno-associated virus (AAV) delivery of bNAbs is safe and capable of sustained bNAb expression for over 18 months following a single intramuscular administration. Here, we review key concepts of effector functions mediated by bNAbs against HIV infection and the potential for vectored immunoprophylaxis as a means of producing bNAbs in patients.

## Introduction

Despite the success of pre-exposure prophylaxis (PrEP) and antiretroviral therapy (ART) in reducing HIV incidence in developed countries, the HIV pandemic remains a major burden in developing nations (1). Among the novel interventions that continue to be developed, those employing broadly neutralizing antibodies are among the most promising as potential prevention (2), therapeutic (3) or cure modality *via* elimination of the latent viral reservoir (4). Broadly neutralizing antibodies (bNAbs) are defined by their capacity for potent neutralization of large panels of diverse strains (5–8). Numerous studies in non-human primates (NHP) and humanized mice have explored the potential for passive transfer of various bNAbs to prevent HIV acquisition. Testing of various antibodies has shown that bNAb-mediated prevention can be highly effective, however, at low concentrations a loss of protection against challenge is observed (9–11).

**Figure 1:**
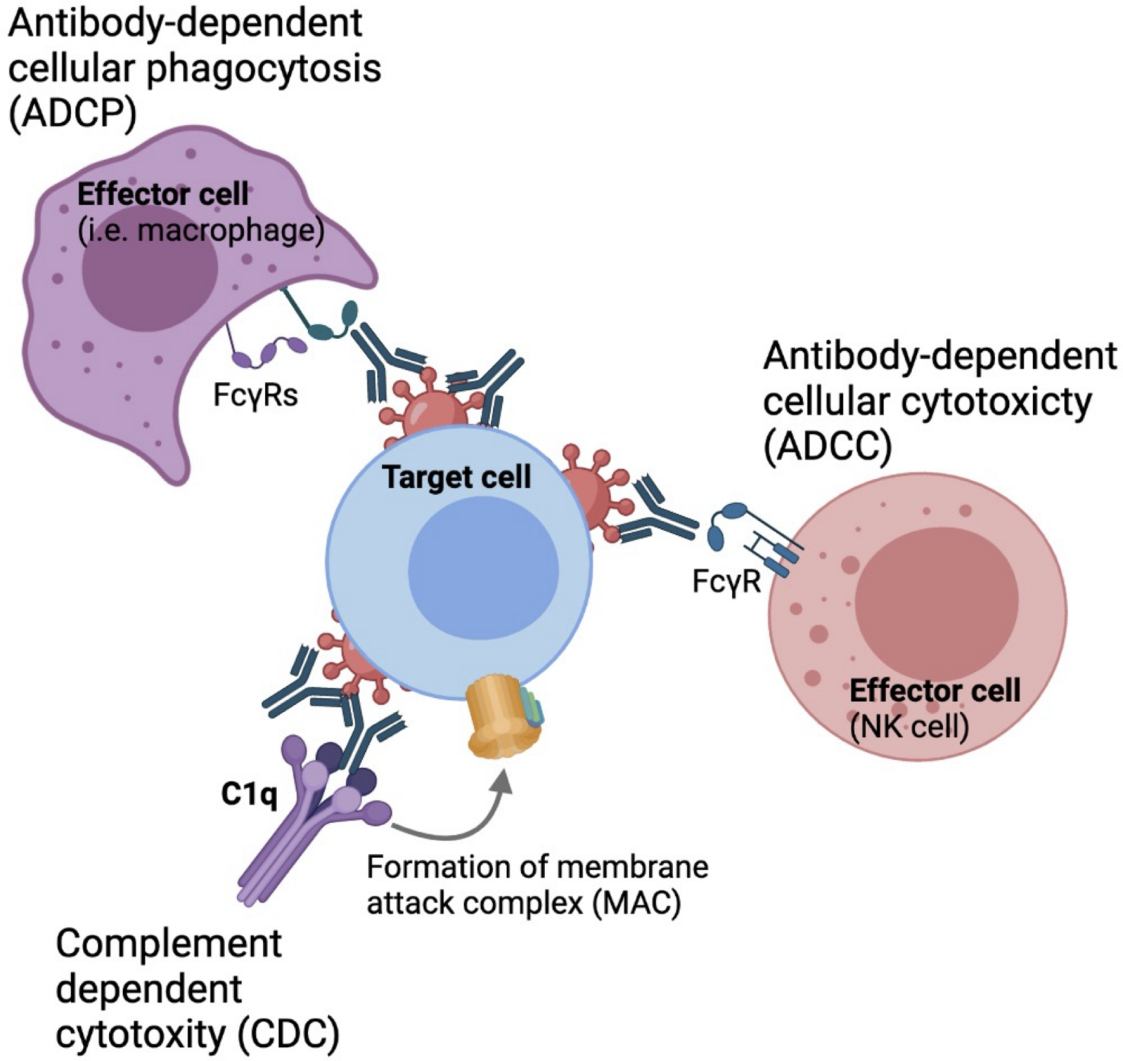
Fc-mediated effector functions. Antibodies can engaged with phagocytes, such as monocytes macrophages and neutrophyls through Fc*γ*RIa and Fc*γ*RIIa to drive antibody-dependent cellular phagocytosis.Nk cells can engaged with antibodies through engagement with Fc*γ*RIIIa to drive antibody-dependent cellular cytotoxicity. Antibodies can also activate the classical complement pathway to derive complement dependent cytotoxicity.

In addition to direct neutralization of virus entry, antibodies are capable of mediating non-neutralizing functions that are important in the context of HIV prevention and viral clearance, such as antibody-dependent cellular cytotoxicity (ADCC), antibody-dependent cellular phagocytosis (ADCP) and antibody-dependent complement deposition (ADCD), through engagement of the Fragment crystallizable (Fc) region with various Fc receptors expressed on the surface of innate cells or complement proteins in the circulation (**Figure 1**) (12–16). Therefore, understanding the parameters that influence bNAb engagement with these innate cell subsets is critical for the development of maximally effective protective and therapeutic strategies.

Recent results from the AMP study, in which patients were passively transferred with VRC01 to prevent HIV acquisition, has shown that transmission risk is increased as antibody concentrations fall (17) (ClinicalTrials.gov: HVTN 703/HPTN 081). To overcome the short-lived nature of passive transfer, we and others have described recombinant adeno-associated viruses (rAAVs) as a delivery modality, termed Vectored ImmunoProphylaxis (VIP), that utilizes a single intramuscular administration to yield sustained expression of a given antibody (18). To maximize packaging capacity and minimize any potential toxicity, all viral sequences are removed with the exception of two 145 base pair inverted terminal repeats. These innovations allow for a full-length antibody sequence to be successfully packaged and efficiently expressed in animal models (19, 20).

Humanized mice are a valuable model in which to test both prophylactic and therapeutic interventions for HIV. These mice are the product of genetic engineering to express human genes or xenografting of immunocompromised mice with stem human cells and tissues. Humanized mouse models have shown long-lasting expression of various HIV-1 bNAbs delivered through VIP resulting in protection from viral challenge (21, 22). Given the pre-clinical success of vectored antibody strategies, a clinical trial testing the safety and efficacy of bNAb delivery using VIP is currently underway with recent results showing up to microgram per mL concentrations in circulation which were sustained for at least 18 months post-administration. In this review, we discuss the role of Fc-mediated effector functions during antibody-mediated protection from HIV particularly at low concentrations and the potential for vectored immunoprophylaxis to harness effector functions to yield durable protection through sustained bNAb delivery.

## Protection From HIV Acquisition by Broadly Neutralizing Antibodies

The first-generation of HIV-1 broadly neutralizing antibodies including b6, 4E10, 2F5, and b12 were described over twenty years ago and showed limited breadth or potency (23–26). Since then, several second generation bNAbs such as PGDM1400, VRC01, and PGT121 have been characterized with far greater potency and breadth (27). BNAbs target distinct sites of vulnerability on the viral envelope; these include the CD4-binding site (CD4bs), the V1V2 loops (V1V2), the V3 loop (V3), the membrane-proximal external region (MPER), and more recently the gp120-41 interface or fusion peptide (28–31). Studies in non-human primates (NHPs) have investigated bNAb-mediated protection from SHIV acquisition using various bNAbs targeting each of these sites, including b12 (13), VRC01 (11, 32), 3BNC117 (CD4bs) (9, 33), PGDM1400, CAP256-VRC26.25 (V1V2) (10), 10-1074 (34) and PGT121 (V3) (9, 35). A recent meta-analysis of passive immunization studies performed by Pegu et al. (32) measured the rates of SHIV infection after a single administration of a given antibody (32). Collectively, these studies showed that antibody serum concentration against the challenge virus was strongly correlated with protection and that antibody inhibitory concentration required to reduce viral infectivity by fifty or eighty percent (IC_50_ and IC_80_, respectively) were also strong predictors of protection. Other work has shown that the protective concentration of an antibody *in vivo* is often 50-200 times greater than the *in vitro* IC_50_ value calculated in an *in vitro* neutralization assay suggesting that the more potent an antibody is, the lower the concentration required to protect (36). Collectively, this work shows that antibody concentration and potency are crucial factors that contribute to protection. However, antibodies are also capable of mediating non-neutralizing effector functions that have been shown to contribute to protection and driving viral clearance (12, 13, 16, 37, 38).

## Contribution of Fc-Mediated Effector Functions in HIV Prevention and Viral Clearance

Innate immune cells such as natural killer cells, monocytes, macrophage and neutrophils express a variety of activating and inhibitory Fc-gamma receptors (FcγRs) which can engage with the Fc-region of antibodies to drive Fc-mediated effector functions (39–41). Four different subclasses of Fc receptors have been defined, including three activating receptors FcγRI, FcγRII and FcγRIIIa, as well as the inhibitory receptor FcγRIIb. These molecules drive antibody-dependent innate functions including ADCC, ADCP, ADCD. ADCC occurs when NK and other innate cells form immunological synapses with a target cell through FcγRIIIa engagement to release perforin and granzyme B. These cytotoxic granules create pores in the membrane of the target cell, causing it to lyse and die (42–44). Phagocytosis is mediated by monocytes, macrophages, and neutrophils, where immune complex-opsonized cells are engulfed by mononuclear phagocytes through engagement of FcγRIa and FcγRIIa. Cross-linking of these FcγRs leads to downstream degradation of these engulfed target cells (45). Additionally, antibodies can also engage the complement system to drive ADCD. Antibodies bound to envelopes expressed by infected cells can form stable hexameric immune complexes that can recruit complement (45). Antibody-mediated complement activation occurs through the classical pathway in which C1q is recruited to antibody-immune complexes (46). This results in the formation of a membrane attack complex (MAC) that leads to lysis of the target cell (47).

The role of Fc-mediated effector functions in HIV prevention in patients has been suggested by the analysis of the partially successful RV144 vaccine trial in Thailand, in which patients were administered a heterologous prime-boost vaccine regimen (48). Analysis of uninfected participants showed that antibodies capable of driving Fc-mediated effector functions were positively associated with protection (14). Two antibody-dependent innate functions that were strong correlates of decreased risk were ADCC and ADCP (49–51). Interestingly non-neutralizing IgG1 and IgG3 subclass antibodies targeting the V1V2 site mediated these effector functions, raising the possibility that there may be an optimal envelope epitopes to target to best drive these Fc functions. Bradley et al. performed a similar study to that of the RV144 trial in non-human primates, however they designed a vaccine cocktail of various gp120s to increase the diversity of antigen seen by the immune system in the hopes of eliciting broadly neutralizing antibodies (52). Analysis of the elicited antibodies also found that ADCC by NK cells was a major correlate of protection in the monkeys. More recent studies investigating the correlates of protection in a phase 1B trial attempting to replicate the RV144 regimen in South Africa (HVTN 097) found that innate immune pathways were highly upregulated, including signatures of ADCC and ADCP however it failed to demonstrate significant protection (53). Collectively, these findings suggest antibodies capable of driving Fc-mediated effector functions may hold promise in future interventions designed to prevent HIV transmission.

In addition to HIV prevention, polyfunctional antibodies have also been found to be associated with HIV control (54). Similar to findings described in RV144 subjects, elite controllers, who maintain low viral loads in the absence of therapy, harbor higher levels of IgG1 and IgG3 antibodies capable of mediating ADCC and ADCP. Antibodies isolated from elite controllers, viremic controllers, infected patients on ART, and infected patients off ART were compared for polyfunctionality and breakdown of IgG isotype. Interestingly, antibodies from elite controllers did not have enhanced polyfunctionality compared to other groups, but there was a higher prevalence of IgG1 and IgG3 subclasses seen in these patients. These antibodies were also able to mediate ADCC, ADCP, ADCD, and antibody-dependent neutrophil phagocytosis (ADNP) at lower serum titers compared to the other groups (54). Additionally, patients harboring antibodies that could mediate ADCC were also more likely to develop antibodies that could drive phagocytosis by both monocytes and neutrophils. Collectively, this data suggests that polyfunctional antibodies may have a significant impact on viral clearance in chronically infected individuals.

Understanding the role effector functions play in both prevention and control in chronic infection has largely focused on non-neutralizing antibodies elicited by patients. Although the vaccine regimen administered in the RV144 trial showed promising results, when an analogous regimen was given in South Africa in the HVTN02 trial it failed to achieve statistically significant protection, demonstrating the need for continued development of alternative strategies. Additionally, a study by Dugast et al. (55) demonstrated that passive transfer of ADCC-inducing non-neutralizing antibodies isolated from elite controllers failed to protect rhesus monkeys from SHIV challenge, suggesting that Fc-mediated effector functions alone are insufficient to protect against viral acquisition (55). In another study, Burton et al. (56) showed that rhesus macaques had limited protection against mucosal SHIV challenge when administered weakly or non-neutralizing antibodies compared to monkeys given a potent bNAb (56). Given these findings, highly potent neutralizing antibodies appear to afford better protection than polyfunctional non-neutralizing antibodies in HIV prevention. However, understanding the capacity for bNAbs to elicit these Fc-mediated effector functions has been of considerable interest as a potential way to harness the polyfunctionality of these antibodies. Additionally, given the promise shown by bNAbs in prevention, there has also been a push to investigate how these antibodies may be employed therapeutically.

A number of groups have independently performed assays designed to measure the protective and therapeutic efficacy of bNAbs across a wide-range of animal models (**Table 1**). Seminal work in this area by Hessell et al. demonstrated the importance of Fc-mediated effector functions of bNAbs in SHIV prevention. Variants of b12, a first-generation CD4bs bNAb, designed to abrogate Fc-interaction and engagement with FcγRs, were passively transferred into rhesus macaques to evaluate the contribution of Fc effector functions against SHIV challenge (13). Interestingly, NHPs that were given b12-LALA, an Fc variant in which mutations were engineered into the Fc region to diminish engagement with FcγRs and abrogate ADCD, ADCC, ADCP, and ADNP, were more susceptible to infection compared to macaques that were administered wildtype antibody. However, recent work by Hangartner et al. (60) performing a similar study with PGT121 found differing results (60). Demonstrating no difference in protection among NHPs given PGT121-WT and PGT121-LALA or PGT121-LALAPG, a variant that further reduces engagement with rhesus FcγRI and rhesus FcγRIIa, thereby reducing Fc-mediated function. Another recent study found similar results also comparing PGT121 and PGT121-LALA to measure protection afforded by Fc effector function in pigtail marques, finding that the neutralization potency of this bNAb renders Fc effector functions partially redundant (61). It may be more difficult to ascertain the role Fc-mediated effector functions play in protection with a highly potent bNAb, such as PGT121, compared to bNAbs that may be less potent, such as b12 where there may be a more clear distinction in the contribution of effector function (61). Given the results of these studies, it is possible that the protective efficacy of some bNAbs do not benefit from Fc-effector functions. As such, additional work will be needed to fully define the protective properties of Fc-mediated functions.

**Table 1:** Protective and therapeutic efficacy of bNAbs *in vivo*.

Reference	Model System	bNAb	Human or Monkey Ab	Dose given (mg/kg)	Passive Transfer or AAV	Route	Challenge Virus	Virus Route	Dose	In vitro IC50	Projected Conc at time of challenge	Isotype	Effector function	Outcome
Moldt et al. (35)	rhesus macaques	PGT121	Human	5	P.T.	I.V.	SHIV-SF162P3	intravaginal challenge	300 TCID50	0.005µg/ml	High: 95µg/ml	IgG1	N.A.	Protection at 5 and 1mg/kg and 3/5 protected at 0.2mg/kg, suggesting that protective serum concentrations for PGT121 is <10µg/ml
				1							Medium: 15µg/ml			
				0.2							Low:1/8µg/ml			
Shingai et al. (57)	rhesus macaques	VRC01	Human	**Against AD8EO:**	P.T.	I.V.	SHIV-DH12-V3AD8	rectal challenge		AD8EO:	AD8EO:	IgG1	N.A.	Neutralizing titers were predictive of protection against both viruses; the higher the antibody conentration the more likely a monkey was to be protected
		NIH45-46		VRC01: 50, 20							VRC01: 188-711µg/ml			
		45-46G54W		PGT121 and 10-10-74: 20, 5, 1, 0.2						V3:0.09-0.15µg/ml,	PGT121: 1.8-267µg/ml			
		45-46m2								CD4bs: 0.14µg/ml-6.36µg/ml	10-1074: 19-289µg/ml' 3BNC117: 215-105µg/ml			
		3BNC117		3BNC117: 5, 1							45-46m2: 2-15µg/ml			
		12A12		45-46m2: 20, 5						DH12-V3AD8 V3:	DH12-V3AD8:			
		1NC9		**Against DH12-V3AD8:**			SHIV-AD8EO				VRC01: 306-395µg/ml			
		8ANC195		VRC01: 30						V3: 0.01-0.16µg/ml	PGT121: 1-282µg/ml			
		10-1074		PGT121, 10-1074 and 3BNC117: 20, 1, 0.2, 0.05							10-1074: 19-290µg/ml 3BNC117: 3-294µg/ml			
		PGT121								CD4bs: 0.39-86.27µg/ml	45-46m2: 2-4µg/ml			
		PGT126		45-46m2: 5										
Pegu et al. (32)	rhesus macaques	2D5	Human	2D5:40	P.T.	I.V.	SHIV-SF162P3	rectal challenge	300 TCID50		2D5: 352µg/ml	IgG1	N.A.	Protection with 2D5 (2/4) despite high concentration, VRC01 against SHIV-SF162P3 afforded complete protection, BalP4 challenges: all monkeys at high and medium doses of VRC01 were protected, at low dose (4/10), 10E8 protected all monkeys at high and medium doses, at low dose (3/6), PG9 protected (4/6) at high dose, (3/6) at medium and no moneys at low dose
		VRC01									VRC01 high 60µg/ml, medium 22µg/ml, low 1.31µg/ml			
		1E9		VRC01, 10E8, PG9: 20, 5, 0.3			BALP4				10E8 high 133µg/ml, medium 31µg/ml, low 1.8µg/ml			
		PG9									PG9 high 32µg/ml, medium 3.7µg/ml, low 0.28µg/ml			
Julg et al. (10)	rhesus macaques	PGDM1400	Human	2	P.T.	I.V.	SHIV-325c	rectal challenge	500 TCID50	PGDM1400=0.037	~0.1-10µg/ml	IgG1	N.A.	CAP256.VRC26 protection at high dose (3/3), medium (3/3), low (3/3), PGMD1400 protection at high dose (4/5), medium (5/5/), low (1/3)
		CAP256-VRC26.25-LS		0.4						CAP256-VRC26.25=0.003				
				0.08										
Balazs et al. (18)	Hu-PBMC mice	2G12	Human	N.A.	AAV	I.V.	NL4-3	I.P. and I.V.	1ng p24		b12: 100µg/ml	IgG1	N.A.	Mice given b12 were completely protected, mice given 2G12, 2F5 and 4E10 were partially protected. Mice expressing varying doses of VRC01 showed partial protection: Mice expressing less than 10µg/ml succumbed to infection but mice expressing >10µg/ml were protected
		b12									2G12: 150µg/ml			
		2F5									2F5: 20µg/ml			
		4E10							10ng p24		4E10: 20µg/ml			
		VRC01									VRC01: 0.1-200µg/ml			
Balazs et al. (21)	BLT humanized mice	b12	Human	N.A.	AAV	I.V.	REJO.c	intravaginal challenge	16ng p24 REJO.c		b12: ~100-300µg/ml	IgG1	N.A.	bNAbs can maintain long lasting expression using VIP, can also reach high concentrations that are protective against repeated mucosal challenge
		VRC01					JR-CSF		50ng p24 JR-CSF		VRC01:~100-300µg/ml			
		VRC07W									VRC07W: ~100µg/ml			
Moldt et al. (58)	rhesus macaques	PGT126	Human	10	P.T.	I.V.	SHIV-SF162P3	intravaginal and rectal	unknown	0.3µg/ml	10mg/kg: 100-125µg/ml	IgG1	N.A.	No difference in protection between either route of challenge, suggesting that there is similar effiacy of bNAb proteection against both primary transmission routes
				0.4							2mg/kg:25µg/ml			
											0.4mg/kg: 4µg/ml			
Rudicell et al. (59)	rhesus macaques	VRC01-LS	Human	0.3	P.T.	I.V.	SHIV-BalP4	rectal challenge	12,800 TCID50	VRC01-LS: 0.028µg/ml	VRC01-LS: 2.5µg/ml	IgG1	N.A.	VRC07-523-LS afforded better protection compared to VRC01-LS, suggesting that a more potent antibody can protect at these lower concentrations
		VRC07-523-LS		0.2						VRC07-523-LS: 0.005µg/ml	VRC07-523-LS: 0.47µg/ml			
				0.05										
Saunders et al. (11)	rhesus macaques	VRC01	Simian	5	P.T.	I.V.	SHIV-BalP4	rectal challenge	unknown	0.019µg/ml	VRC01: 0.1-1µg/ml	IgG1	N.A.	Introducing an LS mutation into the antibody led to elevated antibody levels for a longer period of time and protected against mucosal challenge for up to two months after last antibody administration
		VRC01-LS									VRC01-LS: 2-6µg/ml			
Ko et al. (59)	rhesus macaques	VRC01, VRC01-LS	Human	0.3	P.T.	I.V.	SHIV-BalP4	rectal challenge	unknown	unknown	~20-100µg/ml	IgG1	FcRn and FcγRIIIa binding, ADCC	VRC01-LS affords better protection against viral challenge than VRC01, due to its enhanced binding with FcRn. No detectable difference in the ability to bind FcRIIIa, suggesting that ADCC is intact
		VRC01-LS												
Hessell et al. (13)	rhesus macaques	b12-WT	Human	1	P.T.	I.V.	SHIV-SF162P3	intravaginal challenge	TCID50 10	0.18µg/ml	b12: ~45-70µg/ml	IgG1	b12-WT can mediate effector functions, LALA variant cannot mediate any function	Two-fold difference in hazard ratio between WT and LALA variant number of challenges to infection, effector function appears to play a role in this difference in protection
		b12-LALA									b12-LALA: ~5-55µg/ml,			
Hessell et al. (38)	rhesus macaques	b12-WT	Human	25	P.T.	I.V.	SHIV-SF162P3	intravaginal challenge	300 TCID50	unknown	b12-WT: 562µg/ml	IgG1	C1q and FcγR binding	No difference in protection between b12-WT and b12-KA (8/9 protected), but monkeys given b12-LALA were less protected (5/9)
		b12-LALA									b12-KA: 616µg/ml			
		b12-KA									b12-LALA: 534µg/ml			
Bournazos et al. (37)	Luciferase reporter mice transduced with AdV hCCR5-A2-hCD4	3BNC117	mouse-human chimeric (human bNAbs with mouse constant region heavy chains)	200µg	P.T.	S.C.	HIV-YU-2 Cre pseudovirus	I.V.	unknown	3BNC117: 0.021	unknown	mIgG2a and mIgG1 D265A	FcγR binding as a surrogate for Fc effector function	mIgG1 and mIgG1 D265A (Fc-null) variants of bNAbs had higher rates of infection compared to mIgG2a (intact Fc function) variants of all bNAbs, suggesting that Fc-mediated effector functions play a role in protection
		Jan-74								1-74: >50				
		3BCN60								3BNC60: 0.018				
		Jan-79								1-79: 24.8				
		3BC176								3BNC176: 1.278				
		PGT121								PGT121: 0.44				
		PG16								PG16: 0.8				
Bournazos et al. (37)	NRG humanized mice	3BNC117-WT	Human	100µg/ml (high dose)	P.T.	S.C.	HIV-YU-2	I.V.	57.5ng p24	0.021µg/ml	>10µg/ml	IgG1	FcγR binding as a surrogate for Fc effector function	Mice given 3BNC117-GASDALIE (Fc enahncing) exhibited lower rates of infection compared to WT and 3BNC117-GRLR (Fc-null)
		3BNC117-GRLR		20µg/ml (low dose)										
		3BNC117-GASDALIE												
Julg et al. (10)	rhesus macaques	3BNC117, PGT121	Human	10	P.T.	I.V.	SHIV-327c	rectal challenge	300 TCID50	PGT121=0.11µg/ml	~50-150µg/ml	IgG1	ADCP and CDC	PGT121 protected monkeys at both doses (high dose 4/4, low dose 2/2), 3BNC117 did not protect at low dose (0/3) and only protect 1/4 monkeys at high dose, no dfference in mediating effector function
				2						3BNC117=0.84µg/ml				
Hangartner et al. (60)	rhesus macaques	PGT121	Human	1	P.T.	I.V.	SHIV-SF162P3	intravaginal challenge	300 TCID50		PGT121: 5-10µg/ml	IgG1	ADCP and ADCC	No difference in protection between PGT121 and PGT121-LALA, suggesting that effector function for this antibody maynot contribute to protection against this given virus
		PGT121-LALA,									PGT121-LALA: 5-18µg/ml			
		PGT121-LALAPG									PGT121-LALAPG: 5-20µg/ml			

N.A., not applicable; S.C., subcutaneous

In addition to prevention, Fc-mediated effector functions of bNAbs have also been investigated in the context of driving viral clearance of established HIV infection. Recently, Asokan et al. measured the contribution of bNAb-mediated effector function in chronically infected rhesus macaques. SHIV-infected monkeys were treated with a CD4bs-directed bNAb, VRC07-LS, or variants harboring Fc mutations that either enhanced or diminished engagement with FcγRs. Using this panel of mutant antibodies, they determined that innate effector functions, such as ADCC, ADCP and complement fixation contributed approximately 21% of the observed rate of viral clearance (16). Interestingly, this study did find that VRC07-LS with Fc mutations to enhance FcγR binding led to NK cell death one hour after bnAb infusion, likely due to FcγRIIIa cross-linking on the cell surface and driving necroptosis, in turn leading to reduced ADCC. It is possible that over-optimization of an antibody may have detrimental effects early on in delivery, and this should be taken into consideration if these antibodies are to be used therapeutically.

Humanized mice have also been an effective model in which to study the role of antibody effector functions (12, 37, 62). Similar to NHP studies, several groups have investigated the protective effects of various bNAbs and their ability to drive effector functions in the context of both HIV prevention and viral clearance during infection. In one study, humanized NOD *Rag1^-/-^Il2g^null^* (NRG) mice given the CD4-binding site targeting bNAb 3BNC117 and challenged with HIV were found to have enhanced viral clearance 24 hours-post challenge compared to the control mice given a non-specific antibody (37). 3BNC117 has also been used to measure the role of innate immunity in ART treated humanized NRG mice (62). Mutations were introduced into the Fc-region of the antibody to abrogate binding to both murine and human FcγRs. HIV-infected mice taken off ART and given Fc-null (3BNC117-GRLR) antibody exhibited slower viral clearance as compared to mice given the wild-type antibody and similar clearance to control mice given a non-specific antibody. These studies implicate innate cells through Fc engagment in viral clearance in humanized mice. Other work has also aimed to measure the contribution of Fc-mediated viral clearance during early infection (12). In this study, a bi-specific bNAb composed of 3BNC117 and PGDM1400 was administered to infected mice. Another group of infected mice were given the same bNAb harboring mutations in the Fc-region to prevent FcγR engagement and downstream effector functions. Results showed that innate effector functions contributed 25-31% of the antiviral activity seen in humanized mice while 75% was due to antibody neutralization.

Considerable effort has been made to determine whether there are optimal epitopes on the viral envelope that may facilitate these activities. BNAbs targeting each of the major epitopes have been assessed for their ability to mediate innate functions, including ADCC, ADCP, and antibody-dependent complement mediated cell lysis (ADCML) against diverse viruses that span various clades (63, 64). Interestingly, while neutralization potency does not appear to predict effector function, there is a weak correlation between antibody binding and effector function (63, 64). Other groups have confirmed these findings, showing that neither neutralization potency nor breadth are strong predictors of effector activity (64–68). Instead they have shown that binding avidity is better correlated with function (64–68). As of yet, there is no clear consensus of which target epitope for bNAbs best mediates Fc-driven functions. Instead, it has been suggested that modes of binding by non-neutralizing antibodies can have a dramatic effect on the resulting potency of the downstream effector function such as ADCC (67). Two monoclonal antibodies targeting the C1 and C4 gp120 regions with similar antigen affinities exhibit markedly different responses in driving ADCC (67). Interestingly, when crystal structures were determined for each of the antibodies with the corresponding antigen, they showed that antibody orientation on the bound antigen may have enhanced formation of an immune complex, resulting in increased potency of downstream innate immunity (67). The angle of approach for a given bNAb likely affects how these immune complexes can form and engage with FcγRs (67). Additionally, numerous structural studies have elucidated FcγR-Fc interactions to determine the precise mechanism of antibody binding to FcγRs (40, 69–72). Antibody-antigen structures have also provided insight on Fc presentation and angle of antibody binding to an antigen may influence optimal FcγR engagement to lead to downstream effector function, such as ADCC (73). There is still much work to be done to understand how modes of antibody engagement can drive effector functions across diverse viral strains and how antibodies function post antigen-binding.

Other avenues of investigation have looked to optimize bNAbs to elicit more potent Fc effector function. One potential approach has been the use of bNAbs expressed as different isotypes that have enhanced effector function, such as IgG3. Recent work by Richardson et al. (74) compared IgG1 and IgG3 variants of V2-specific bNAbs CAP256-VRC26.25 and CAP256-VRC29 to measure how isotype contributed to potent Fc effector functions (74). The group was able to isolate these two bNAbs from an HIV-infected individual that had mounted a potent V2-specific response and elicited high levels of IgG3 antibodies that significantly contributed to the total effector function activity measured in the serum (74). These two bNAbs, CAP256-VRC26.25 and CAP256-VRC29, were constructed as IgG3 variants using different IGHG3 alleles to measure Fc function and neutralization. They also expressed these bNAbs as IgG1 isotypes and found reduced neutralization and Fc function compared to the IgG3 variants but no difference in antigen binding. They found IgG3 potency was likely due to its longer hinge length than that of IgG1. Therapeutically, however, employing IgG3 in patients may pose a challenge as the affinity for the neonatal receptor FcRn is drastically reduced compared to IgG1, leading to a far shorter half-life (75). However, recent work demonstrated that alteration of the hinge length of IgG1 and IgG3 bNAbs VRC01 and 447-52D contributed to Fc mediated effector functions (76). To make the IgG1 variants, exons derived from IgG3, including exon a which encodes the upper and core hinge regions, and exons b-d that encode the core hinge repeat sequence were sequentially added, resulting in hinge variants up to 5 times longer than wild-type IgG1. They repeated the same hinge alteration for IgG3, and included the IgG1 hinge length as an additional variant. When measuring phagocytosis by all these variants, they found that a longer hinge length significantly enhanced the Fc effector function, suggesting that the length can lead to a more potent response. In addition, *in vivo* stability of hinge variants of IgG1 were measured and the rate of plasma decay in mice was similar to that of the wild-type, suggesting that such alterations may be viable approaches to harness Fc mediated effector function by bNAbs.

In addition to understanding how hinge length, isotype and subclass contribute to optimal Fc effector function, considerable effort has identified point mutations in the Fc region capable of enhancing FcγR engagement and downstream function (77). In 2001, Shields et al. mapped the binding site of IgG1 for FcγRs, including FcγRII, FcγRIIIa and FcRn by mutagenesis of an IgG1 antibody. From these variants, specific point mutations in the Fc region of the antibody emerged that could enhance or diminish binding to the FcγRs described above (78). One modification, S298A/E333A/K334A, showed enhanced binding to FcγRIIIa but decreased binding to FcγRIIa, resulting in improved ADCC response. Further work has elucidated alternative Fc modifications that have led to enhanced FcγRIIIa binding and ADCC, including S239D/I332E, S329D/A330L/I332E, G236A/S239D/I332E and F243L/R292P/Y300L/V305I/P396L (79–81). Other Fc modifications have led to increased FcγRIIa binding and ADCP, including G236A, G236A/A330I/I332E, and G236A/S239D/A330L/I332E (80, 82). Some mutations have led to both enhanced ADCC and ADCP, including S239D/I332E, S239D/A330L/I332E and G236A/S239D/I332E. In addition to introduction of Fc point mutations, afucosylation of IgG1 antibodies led to dramatically increased ADCC (83). Minimal alterations to antibodies can maximize downstream Fc functionality, and these modifications can be used therapeutically to improve bNAb-mediated protection.

Given the potential for bNAb-mediated protection in HIV prevention and viral clearance, the question of implementation has come to the forefront. The AMP clinical trials utilized passive transfer of VRC01, a CD4bs bNAb, to measure HIV prevention in different populations, including HIV-uninfected men who have sex with men (MSM), transgender men who have sex with men, and sexually active women in sub-Saharan Africa (17, 84–86) ClinicalTrials.gov: NCT02716675 (17, 84–86). The results from these trials have been promising, with excellent safety and tolerability to bNAb administration and reduced transmission of sensitive strains. However, one drawback to passive immunization is the need for constant re-administration in order to maintain steady-state bNAb concentrations. This requirement poses significant challenges to feasibly scale and implement as a widespread prophylaxis. In order to overcome this challenge, other methods of delivery can be employed, such as vector-mediated delivery. Adeno Associated Virus (AAV) based gene replacement therapies have been used to treat a variety of diseases, and recent advances in AAV technology have demonstrated sustained bNAb expression in humans in an ongoing clinical trial.

## Utilizing AAV-Delivery of Broadly Neutralizing Antibodies

AAVs are non-enveloped viruses that belong to the Parvoviridae family (85, 86). AAVs are unable to replicate on their own, and as such require a helper virus, such as an adenovirus or herpesvirus to productively replicate within cells (87–90). They are composed of an icosahedral protein capsid surrounding a single stranded DNA genome of approximately 4700 base pairs. The natural AAV genome consists of a *rep* and *cap* gene flanked by two inverted terminal repeats (ITRs) (91). The *rep* gene encodes proteins necessary for virion assembly, including Rep78, Rep68, Rep52 and Rep40. The *cap* gene encodes for three capsid proteins, VP1, VP2 and VP3 and the assembly activating protein (AAP) and membrane-associated accessory protein (MAAP) in alternative reading frames (87, 92–97). AAV virions are comprised of 60 VP subunits, and each subunit has nine variable regions that dictate tropism and intracellular trafficking (91). Currently, there are more than 100 AAV serotypes identified that all differ in primary receptor usage and tissue tropism (98). During infection, AAVs bind to receptors on target cells which trigger endocytosis into endosomes from which they escape and traffic to the nucleus. Here, the inverted terminal repeats (ITRs) present on either end of the genome self-prime second strand synthesis, which is the rate-limiting step prior to gene expression (99–102). The ITRs are the only requirement for packaging DNA into the capsid (103) As a consequence, recombinant AAV vectors have approximately 96% of their genome removed, including all viral coding sequences. This allows for greater packaging capacity and lowers the potential for viral toxicity (91). In place of these coding sequences, a transgene of interest, up to approximately 4,500 base pairs, can be introduced between the ITRs (104). Following transduction, these recombinant viral genomes form head-to-tail concatemers within the nucleus, where they persist as non-integrated episomal genomes (105, 106).

To date, AAV gene therapies have been successful in treating diseases in the therapeutic areas of ophthalmology, neurology, hematology, metabolic and musculoskeletal disorders (99, 107). This platform can also be used for delivering biological therapeutics for chronic infectious diseases, such as HIV, through delivery of small molecule inhibitors, immunoadhesins, or HIV broadly neutralizing antibodies. This platform has the benefit of enabling specific gene-encoded antibodies to be delivered, representing a viable approach to incorporating Fc-enhancing mutations to improve innate immune functionality of the delivered protein. In 2002, Lewis et al. demonstrated the use of a dual promoter rAAV vector to express full-length b12 in Rag1^-/-^ mice (20). Subsequent enhancements were made to the viral vector by Fang et al. improved packaging and cleavage of these antibodies *in vivo* using a picornavirus 2A self-processing peptide in order to express both the heavy and light chains of an antibody from a single open reading frame (19). A furin cleavage site was also introduced between the C-terminus of the heavy chain and N-terminus of the 2A sequence to enable removal of remaining 2A residues (108). These alterations led to significant improvement in antibody expression, resulting in concentrations well over 1mg/ml in serum.

Johnson et al. first demonstrated the efficacy of a self-complementary AAV (scAAV) vector that expressed SIV-specific immunoadhesins composed of recombinant antibody fragments (109). They administered one intramuscular injection of this scAAV1 into rhesus macaques, resulting in serum expression of the immunoadhesins four weeks after administration. Our lab utilized AAV serotype 8 capsid to express a full-length HIV bNAb *in vivo*. Enhancements were made to the vector, including designing a novel promoter that combined the CMV enhancer and chicken β-actin promoter followed by an artificial intron containing the ubiquitin enhancer region (18). These changes led to significantly improved antibody expression which enabled this improved vector system to deliver various HIV bNAbs targeting different viral epitopes and measured protection against repeated HIV mucosal challenge in humanized mice (21). This system has also been used to deliver CAP256-antibodies and CAP228-antibodies in immunocompetent mice, both of which target the V2 loops and have been shown to mediate ADCC (110). NHP studies have also demonstrated expression of a simianized form of VRC07, a CD4bs-directed bNAb, and anti-SIV neutralizing antibodies, ITS01 and ITS06.02 (111, 112). As a result of these promising proof-of-concept animal studies, two separate Phase I clinical trials were initiated to test VIP in humans.

**Figure 2:**
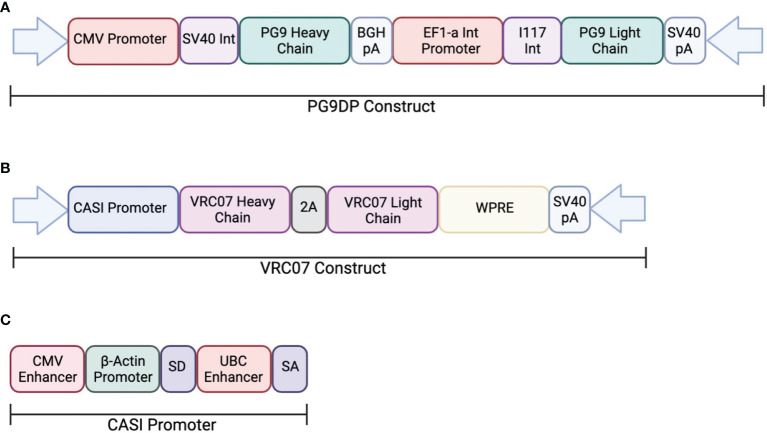
Transgene construct used in clinical trials testing AAV delivery of bNAbs. **(A)** The dual promotor transgene cassette was used to expressed PG9 in an rAAV-1 vector. The heavy chain is expressed the CMV promoter while the light chain is expressed by the EF1a promoter. **(B)** The transgene cassette used to express VRC07 in an rAAV-8 vector. Both heavy and light chains are expressed through the CASI promotor shown in **(C)**.

In one study, AAV was used to deliver PG9, a V1V2-directed bNAb, chosen for its potent neutralization capacity (ClinicalTrials.gov: NCT01937455) (113). This clinical trial looked at the safety and efficacy of PG9 delivery using an rAAV-1 vector. The PG9 heavy and light chains were codon optimized to increase expression in the rAAV vector. Additionally, a 21-amino acid synthetic signal peptide was included in order to improve secretion into the serum. The transgene consisted of a dual promoter system to independently express the variable heavy and variable light chains of the IgG1 antibody. The heavy chain was expressed under a CMV promoter and the light chain was expressed by the EF1a promoter (**Figure 2**). Healthy, non-HIV infected, men aged 18-45 were administered rAAV1-PG9DP through intramuscular injection. Results showed that the AAV caused no harmful side effects to the volunteers, and muscle biopsies showed detectable PG9 by RT-PCR as well as IgG within muscle cells and extracellular tissues by immunohistochemistry. However, PG9 was not detectable in the serum by ELISA, although it is important to note that the limit of detection in the assay was 2.5µg/ml. In support of these findings, no HIV neutralizing activity was detectable in the sera. Importantly, they observed anti-drug antibody responses in many of the participants of the study, suggesting that these may have limited the potential for PG9 expression (113).

Independently, a second on-going clinical trial by the NIH is testing the safety and efficacy of VRC07 delivery through an rAAV8 viral vector to HIV-infected adults aged 18-60 (AAV8-VRC07; ClinicalTrials.gov: NCT03374202). The vector design used in this study was analogous to those described in previous papers from our laboratory and include the CASI promoter and optimized F2A-containing VRC07 transgene (**Figure 2**). Recent results from this clinical trial presented at the 2021 Conference on Retroviruses and Opportunistic Infections meeting showed detectable expression of VRC07 antibody as high as 1µg/mL in circulation, with several patients maintaining VRC07 concentrations well over a year after administration. In contrast to results from the earlier study, VRC07 neutralizing activity was detected in trial participants, suggesting that antibodies produced as a result of VIP retained their activity *in vivo*. This trial represents the first demonstration of long-lived systemic production of a broadly neutralizing antibody in humans, providing strong evidence for the potential of vectored antibody delivery. Despite this success, some participants developed anti-drug antibodies against the VRC07 antibody variable regions, leading to loss of antibody expression. As such, additional improvements to the AAV transgene may be necessary to enable widespread use of VIP for HIV prevention. Looking forward, this system could be utilized for delivery of optimized bNAbs that contain altered Fc-regions with hinge length changes or even different isotypes to improve FcγR binding. The use of such optimized transgenes has the potential to reduce the concentrations of bNAbs necessary to prevent HIV acquisition.

## Discussion

Broadly neutralizing antibodies have been shown to be promising candidates for HIV prevention, therapy and possibly cure. Passive administration of bNAbs have demonstrated that antibody concentration and potency are important parameters correlated with protection against HIV acquisition. Other non-neutralizing functions, including ADCC, ADCP and ADCD have also been shown to be critical in this context. As a result, there is growing interest in understanding whether there are optimal viral epitopes that antibodies can target to effectively elicit protective responses. Early findings from the RV144 vaccine study suggested that non-neutralizing antibodies targeting the V2 site were implicated in preventing disease acquisition. However, when bNAbs targeting different sites of vulnerability were tested for their ability to mediate these same non-neutralizing functions, results were variable and largely dependent upon the challenge virus. Further work is needed to understand if there is an optimal site that can facilitate these functions across a wide range of diverse strains. Additionally, several *in vivo* studies measured the contribution of these effector functions in protection, but questions remain as to contributions of individual functions, such as ADCC, to mediating protection. Future work could help to identify which effector functions contribute most to bNAb-mediated protection, and armed with this improved understanding, specific Fc mutations or hinge length modifications could be made to the antibody to improve therapeutic outcomes. These are especially relevant in the context of vectored antibody delivery, which represents a promising approach capable of integrating advances made in our understanding of antibody-mediated innate immune function into future clinical products. However, transduction efficiency is relatively low as compared to pre-clinical models and there appears to be a significant proportion of recipients who mount an immune response against the antibody transgene. Future studies will be needed to improve the consistency of antibody delivery using this system, including optimizing the vector to improve antibody concentrations and minimizing anti-drug antibodies elicited in response to existing rAAV vectors.

## Author Contributions

MP and AB conceived of and wrote the manuscript. All authors contributed to the article and approved the submitted version.

## Conflict of Interest

The authors declare that the research was conducted in the absence of any commercial or financial relationships that could be construed as a potential conflict of interest.

## Publisher’s Note

All claims expressed in this article are solely those of the authors and do not necessarily represent those of their affiliated organizations, or those of the publisher, the editors and the reviewers. Any product that may be evaluated in this article, or claim that may be made by its manufacturer, is not guaranteed or endorsed by the publisher.
